# Prevalence of Postoperative Pain Following Hospital Discharge: Protocol for a Systematic Review

**DOI:** 10.2196/22437

**Published:** 2020-12-04

**Authors:** Rex Park, Mohammed Mohiuddin, Ramiro Arellano, Esther Pogatzki-Zahn, Gregory Klar, Ian Gilron

**Affiliations:** 1 Department of Anesthesiology and Perioperative Medicine Kingston General Hospital Queen's University Kingston, ON Canada; 2 Department of Anesthesiology, Critical Care Medicine and Pain Therapy University Hospital Muenster Muenster Germany

**Keywords:** pain, postoperative, prevalence, ambulatory, postdischarge, systematic review, epidemiology

## Abstract

**Background:**

Pain is one of the most common, feared, and unpleasant symptoms associated with surgery. However, there is a clear gap in patient care after surgical patients are discharged from hospital, resulting in poorly controlled postoperative pain. Inadequate pain management after discharge can have detrimental effects on quality of life and lead to the development of chronic postsurgical pain. The severity of postoperative pain before discharge is well described, but less emphasis has been placed on assessing pain at home after hospital discharge.

**Objective:**

The objective of this review is to summarize the prevalence of moderate-to-severe postoperative pain within the first 1 to 14 days after hospital discharge.

**Methods:**

A detailed search of epidemiological studies investigating postoperative pain will be conducted on MEDLINE and EMBASE from their inception until the date the searches are run. The primary outcome will be the proportion of patients reporting moderate-to-severe postoperative pain at rest and with movement within the first 1 to 14 days after hospital discharge. The secondary outcomes will include a comparison of postoperative pain after discharge between patients who underwent ambulatory and inpatient surgery, and adverse outcomes attributable to poor pain control after hospital discharge (eg, readmission to hospital, emergency room or other unplanned medical visits, or a decrease in quality of life).

**Results:**

The protocol has been registered in PROSPERO (registration number CRD42020194346). The search strategies for MEDLINE and EMBASE have been completed. The final results are expected to be published in May 2021.

**Conclusions:**

This systematic review is expected to synthesize evidence describing the prevalence of postoperative pain after hospital discharge. Available epidemiological evidence may help inform the magnitude of the problem of postoperative pain at home after hospital discharge.

**Trial Registration:**

PROSPERO International Prospective Register of Systematic Reviews CRD42020194346; https://www.crd.york.ac.uk/prospero/display_record.php?RecordID=194346

**International Registered Report Identifier (IRRID):**

PRR1-10.2196/22437

## Introduction

### Background

The global volume of surgery is large and growing, with approximately 312.9 million operations performed in 2012 [1]. Based mostly on in-hospital evidence, data suggest that up to 80% of patients experience pain after surgery, with over 70% of these patients describing their pain as moderate to severe [2,3]. With advances in surgical and anesthetic techniques and attempts to reduce costs and hospital-acquired morbidity, the duration of hospital stays after surgery continues to decrease [4,5]. In fact, it is estimated that ambulatory surgeries constitute over 60% of all surgeries in the United States, as well as in other countries [6-8]. Shorter hospital stays after surgery shift the onus of adequate pain management from the hospital staff to the patient and their family caregivers. However, discharge instructions provided to patients regarding pain management self-care may be inadequate, unclear, or forgotten by the patient, potentially explaining previous reports of higher pain levels (moderate to severe) following hospital discharge compared with time spent in hospital [4,9].

Adequate pain control after surgery is imperative for successful recovery, and poorly controlled pain can have devastating impacts on patients’ physical functioning, mental health, relationships, and productivity [10,11]. In addition to causing unnecessary suffering, the pain-induced release of catecholamines can cause cardiac complications such as myocardial ischemia and cardiac failure [12]. Pain can also result in chest and abdominal wall splinting, resulting in hypoxemia and alveolar collapse [12]. These complications are associated with an increased economic burden resulting from increases in readmissions, emergency room visits, and the workload of community staff [7,13]. Furthermore, undertreated acute postoperative pain increases the risk of developing chronic postsurgical pain (CPSP) [14,15]. CPSP is estimated to affect 10% to 40% of surgical patients, and given the growing burden of surgical diseases and global volume of surgery, the negative impacts of CPSP are large [16,17]. CPSP is similar to other chronic pain conditions and thus is associated with a severe symptom burden and large economic impact and requires a comprehensive multidisciplinary approach to treatment [18-20].

The goal of relieving acute postoperative pain after hospital discharge must be carefully balanced with the competing goals of managing the adverse effects of analgesic treatments and minimizing other risks, such as the development of persistent opioid use [17]. In addition to acetaminophen and nonsteroidal anti-inflammatory drugs (NSAIDs), opioids are the mainstay of postoperative pain management [21]. As has been long recognized, adverse effects of commonly used nonopioid analgesics (eg, hepatoxicity with acetaminophen; bleeding, impaired bowel anastomosis healing, and renal toxicity with NSAIDs) necessitate careful prescribing and follow-up, and may limit their use and consequently their opioid-sparing effect [21]. With respect to postoperative opioids after hospital discharge, reports suggest that they are frequently prescribed in excess, with potentially inadequate instructions and prescriber follow-up [18,22]. This is particularly concerning given reports of high rates of persistent opioid use after surgery, even in opioid-naïve individuals [23-25]. In addition to these clinical concerns surrounding acute pain management, postsurgical patients may also experience surgical complications (eg, bleeding, and wound infection or dehiscence) and other postoperative cardiorespiratory problems. Since perioperative clinicians (eg, surgeons and anesthesiologists) are generally more focused on higher acuity in-hospital patient care and general practitioners may be uncomfortable managing complex postsurgical patients while they’re recovering at home, the early postdischarge postoperative period may be a vulnerable period, leaving patients’ pain inadequately managed.

Appropriate pain management for surgical patients after hospital discharge is of great importance. The majority of studies focusing on postoperative pain have been conducted on inpatients or ambulatory surgery patients before discharge, whereas the period after discharge appears to be much less investigated. Thus, we aim to perform a systematic review to investigate this period for patients in regard to postoperative pain to quantify the extent of this problem and identify future research needs.

### Objectives

The objective of this review is to synthesize the available evidence on the prevalence of moderate-to-severe postoperative pain within the first 1 to 14 days after hospital discharge and compare the findings in patients who underwent ambulatory surgery (same day) with those who underwent inpatient surgery (at least 1-night hospital stay). We will also describe the adverse outcomes that participants experienced that are attributable to poor pain control after hospital discharge, including readmission to hospital, emergency room or other unplanned medical visits, and decreased quality of life.

## Methods

### Guidelines

This protocol was developed in accordance with the Preferred Reporting Items for Systematic Reviews and Meta-Analyses Protocols (PRISMA-P) checklist [26] (Multimedia Appendix 1) and has been registered in the PROSPERO database (registration number CRD42020194346). The systematic review will be carried out in accordance with the Preferred Reporting Items for Systematic Reviews and Meta-Analyses (PRISMA) guidelines [27] and the Meta-analyses Of Observational Studies in Epidemiology (MOOSE) checklist [28].

### Sources of Evidence

We will conduct a detailed search on MEDLINE and EMBASE from their inception until the date the searches are run. The search will include terms relating to postoperative pain, the time frame following hospital discharge, and search filters for epidemiological studies. The search strategies for MEDLINE (Multimedia Appendix 2) and EMBASE (Multimedia Appendix 3) were developed in consultation with a librarian with expertise in literature searches. We will also review the bibliographies of any studies identified for relevance. We will not be contacting experts or searching the grey literature to identify additional studies.

### Types of Studies

The review will include postoperative pain epidemiological observational studies with postsurgical patients that assessed postoperative pain at home (ie, after hospital discharge). Studies with fewer than 100 participants will be excluded.

### Types of Participants

We will include studies with adults aged 18 years and over that underwent a surgical procedure.

### Data Collection, Extraction, and Management

Two trained reviewers (RP and MM) will independently evaluate studies for eligibility. Screening will be performed on titles and abstracts using Covidence software [29]. Citations will be stored in EndNote software (Clarivate Analytics). Full-text screening will be performed on citations felt to be potentially eligible. Disagreements between reviewers will be resolved by discussion and consensus, and if necessary, a third reviewer will be consulted (IG). The screening and selection process will be presented using a PRISMA flowchart, and reasons for exclusion will be reported.

Data from included studies will be extracted using standardized extraction forms specifically designed for this review. These forms will capture information about the surgical procedure (eg, total knee arthroplasty), total number of participants before and after dropouts, patient inclusion and exclusion criteria, patient characteristics (eg, age, medical diagnoses), time points for pain intensity measurements, primary and secondary outcome measures, and other study characteristics (eg, date of publication).

### Primary Outcome

Our primary outcome will be the proportion of patients reporting moderate-to-severe postoperative pain at rest or with movement, or both, within the first 1 to 14 days after hospital discharge. We chose this time frame because the first couple of weeks after surgery are most commonly associated with pain of the highest severity and most functional consequences [30,31]. We will preferentially use 4/10 (numerical rating scale), 40/100 (visual analog scale), or ≥moderate pain (category scale) as the bottom threshold for moderate pain. However, if those specific data are not available and if a study provides pain prevalence estimates using their own definition of moderate pain, we will use those data as provided.

### Secondary Outcomes

Our secondary outcomes will include the following: (1) a comparison of the proportion of participants reporting moderate-to-severe postoperative pain within the first 1 to 14 days after discharge between those who underwent ambulatory surgery (same day) and those who underwent inpatient surgery (at least 1-night hospital stay); and (2) adverse outcomes experienced by participants within the first 1 to 14 days after discharge that are attributable to poor pain control, including readmission to hospital, emergency room or other unplanned medical visits, and decreased quality of life.

### Analysis of Outcomes

Only similar studies (eg, similar surgical procedures, similar postoperative days, and outcomes measured) will be combined for analysis in order to avoid clinical heterogeneity. Extracted data will be compiled in Microsoft Excel for analysis. Analysis will be performed using Review Manager (RevMan) software (version 5.3; The Nordic Cochrane Centre, The Cochrane Collaboration). We plan to use a random-effects model for meta-analysis for primary and secondary outcomes if it is deemed appropriate to combine heterogenous studies.

If inappropriate to combine studies, a descriptive approach will be used to report the primary and secondary outcomes. The findings will be organized and presented by type of surgical procedure.

### Assessment of Risk of Bias in Included Studies

Risk of bias for each study will be independently assessed by 2 reviewers (RP and MM). We will use the risk-of-bias tool for prevalence studies developed by Hoy et al [32], which includes 10 items plus a summary assessment (Multimedia Appendix 4). Items 1 to 4 assess the external validity of the study, and items 5 to 10 assess the internal validity. Disagreements between reviewers will be resolved with discussion and consensus. If necessary, a third reviewer (IG) will be consulted.

### Assessment of Heterogeneity

Heterogeneity will be evaluated using the *I*^2^ statistic. When the *I*^2^ value is higher than 50%, we will consider possible explanations for it.

### Sensitivity Analysis

We plan to conduct a sensitivity analysis where we combine data from all studies that focused on any degree of postoperative pain after discharge rather than only the studies that focused on moderate-to-severe pain.

## Results

The protocol has been registered in PROSPERO (registration number CRD42020194346). The search strategies for MEDLINE and EMBASE have been completed. The final results are expected to be published in May 2021.

## Discussion

The postoperative period after hospital discharge is a particularly vulnerable time in patients’ clinical trajectory. Patients continue to experience unacceptable levels of pain after discharge [2,3]. Given the dynamic nature of postoperative pain, provision of optimal pain management requires ongoing assessments and adjustments of pain management plans [30]. However, after hospital discharge, this is difficult for surgeons, general practitioners, and other community health care workers because of time constraints. There may also be limited transitional pain services available and, as a result, patients are required to take on a large responsibility for their own pain management. Patients may also be overprescribed opioids with insufficient instructions, increasing their risk of opioid misuse, abuse, and addiction [22,33]. To complicate matters, some general practitioners lack the comfort in weaning their patients off opioids, which may partly be due to the limited research available on safe and effective postoperative opioid weaning strategies [23,34]. On the contrary, physicians responding to the opioid crisis by minimizing opioid prescriptions or tapering their patients off opioids without other nonopioid strategies to help control their pain may put postsurgical patients at risk of pain undertreatment and the development of CPSP. Additionally, patients may not have assistance at home, where mobility obstacles are plentiful and when pain intensity may be at its greatest as hospital-administered multimodal analgesics, including regional analgesia, are wearing off [2].

Poor postoperative pain control after discharge may cause unnecessary discomfort, the development of CPSP, and various pathophysiological complications of pain. In addition to improving clinical outcomes, prevention and effective management of pain after discharge may reduce hospital readmissions, unsafe analgesic use, and the burden on community-based health care providers [35]. However, research evaluating the magnitude of this problem or the adequacy of pain assessment of postsurgical patients who are recovering at home is limited. This is especially true when compared with the evidence of inpatient postoperative pain. Various and diverse sources of evidence about this topic are accumulating and vigilant evaluation and synthesis are needed. Thus, this review aims to summarize the prevalence of moderate-to-severe postsurgical pain after hospital discharge. The findings of this review may guide further research evaluating this large gap in patient care after hospital discharge, highlight services in need of more resources and consideration, and improve patient outcomes at home.

## Appendix

Multimedia Appendix 1 Preferred Reporting Items for Systematic Reviews and Meta-Analyses Protocols (PRISMA-P) checklist.

Multimedia Appendix 2 Search strategy developed for MEDLINE.

Multimedia Appendix 3 Search strategy developed for EMBASE.

Multimedia Appendix 4 Risk-of-bias tool for prevalence studies.

## Abbreviations

CPSP: chronic postsurgical pain
MOOSE: Meta-analyses Of Observational Studies in Epidemiology
NSAIDs: nonsteroidal anti-inflammatory drugs
PRISMA: Preferred Reporting Items for Systematic Reviews and Meta-Analyses
PRISMA-P: Preferred Reporting Items for Systematic Reviews and Meta-Analyses Protocols
