# Supplementary material for: Prevalence of Postoperative Pain Following Hospital Discharge: Protocol for a Systematic Review
Source: JMIR Res Protoc. 2020 Dec 4;9(12):e22437. doi: 10.2196/22437 (PMC7748962; doi:10.2196/22437)
Supplement: Multimedia Appendix 2 [file resprot_v9i12e22437_app2.doc]

1 exp Pain, Postoperative/ (40555)

2 Postoperative Care/ (59123)

3 Postoperative Period/ (50102)

4 Postoperative Complications/ (361551)

5 pain*.ab,ti. (680419)

6 exp pain/ (391646)

7 Pain Measurement/ (85071)

8 2 or 3 or 4 (452526)

9 5 or 6 or 7 (838909)

10 8 and 9 (34929)

11 1 or 10 (69294)

12 Patient Discharge/ (29213)

13 postdischarge.mp. (4172)

14 post-discharge.mp. (5057)

15 discharge*.mp. (264552)

16 home*.ab,ti. (483564)

17 or/12-16 (725264)

18 11 and 17 (4719)

19 (prevalence or incidence or epidemiol* or survey or rapid assessment or situation assessment or situational assessment or rar or cohort or surveillance or seroprevalence or seroincidence or seroepidemiol* or screening).mp. or exp epidemiologic methods/ or exp epidemiologic studies/ or exp sentinel surveillance/ or exp seroepidemiologic studies/ or exp cohort studies/ or exp cross-sectional studies/ or exp longitudinal studies/ or exp follow-up studies/ or exp prospective studies/ (7751652)

20 epidemiology.fs. (1646524)

21 epidemiology/ or (incidence or prevalence or epidemiology).ti,ab. (1396320)

22 (incidence or prevalence or epidemiology).ti. (290110)

23 19 or 20 or 21 or 22 (7751652)

24 18 and 23 (3532)

25 24 not (exp animals/ not humans.sh.) (3508)

26 25 not (exp child/ not adult/) (3103)
