# Supplementary material for: Prevalence of Postoperative Pain Following Hospital Discharge: Protocol for a Systematic Review
Source: JMIR Res Protoc. 2020 Dec 4;9(12):e22437. doi: 10.2196/22437 (PMC7748962; doi:10.2196/22437)
Supplement: Multimedia Appendix 3 [file resprot_v9i12e22437_app3.docx]

1 postoperative pain/ (68945)

2 postoperative care/ (96492)

3 postoperative period/ or postanesthesia care/ (217326)

4 exp postoperative complication/ (708945)

5 pain*.ab,ti. (1068698)

6 exp pain/ (1394693)

7 exp pain assessment/ or exp pain measurement/ (179497)

8 5 or 6 or 7 (1778922)

9 2 or 3 or 4 (945701)

10 8 and 9 (165605)

11 1 or 10 (165605)

12 hospital discharge/ (126590)

13 postdischarge.mp. (5277)

14 post-discharge.mp. (11547)

15 discharge*.mp. (494820)

16 home*.ab,ti. (675658)

17 12 or 13 or 14 or 15 or 16 (1120837)

18 11 and 17 (15310)

19 (epidemiolog* or incidence* or prevalence*).ti. (461489)

20 ((epidemiolog* or incidence* or prevalence*) adj12 studies*).ab. (181619)

21 epidemiology.fs. or exp incidence/ or exp prevalence/ or (epidemiol* or inciden* or prevalen*).ti,ab. (3412014)

22 (prevalence or uncidence or burden or trend* or diagnos* or number* or (survey* or cross section* or cross-section* or cohort*)).mp. or exp morbidity/ or exp epidemiologic studies/ (13258726)

23 19 or 20 or 21 or 22 (14025116)

24 18 and 23 (9727)

25 limit 24 to animal studies (58)

26 24 not 25 (9669)

27 limit 26 to child <unspecified age> (809)

28 26 not 27 (8860)

29 limit 28 to (books or conference abstract or conference paper or "conference review" or editorial or letter) (2291)

30 28 not 29 (6569)
